# Levodopa reverse stridor and prevent subsequent endotracheal intubation in Parkinson disease patients with bilateral vocal cord palsy: A case report

**DOI:** 10.1097/MD.0000000000005559

**Published:** 2016-12-16

**Authors:** Chia-Chan Tsai, Meng-Ni Wu, Li-Min Liou, Yang-Pei Chang

**Affiliations:** a Department of Neurology, Kaohsiung Municipal Ta-Tung Hospital, Kaohsiung Medical University; b Department of Neurology, Kaohsiung Medical University Hospital; c Department of Neurology, Kaohsiung Medical University, Kaohsiung, Taiwan.

**Keywords:** dopaminergic therapy, Parkinson disease, respiratory failure, stridor, upper airway obstruction

## Abstract

**Background:**

Respiratory abnormalities are often overlooked; however, because of their potential comorbidity, they must be analyzed to determine the most effective treatment for patients with Parkinson disease (PD). Among various theories on respiratory abnormalities in PD, “upper airway obstruction” and “restrictive respiratory disorders” are 2 of the most accepted etiologies; both appear to be related to basal ganglia dysfunction. Complex vocal cord muscle dysfunction contributes to stridor, which can be a manifestation of nigrostriatal dopaminergic dysfunction. Stridor is a lethal form of upper airway obstruction in PD patients; its most frequent causes are bilateral vocal cord palsy, laryngeal spasms, and dystonia of the supra-laryngeal muscle. Several previous studies have suggested that levodopa administration induces a significant improvement of both lung function and symptoms of parkinsonian syndrome.

**Case Summary:**

We reported a 77-year-old gentleman PD patient admitted for acute levodopa-responsive stridor resulting from bilateral vocal cord palsy. Dopaminergic therapy prevented the need for subsequent endotracheal intubation and tracheostomy treatment.

**Conclusion:**

It is vital to understand that complex vocal cord muscle dysfunction may be related to nigrostriatal dopaminergic dysfunction in PD patients. The strategy of levodopa up-titration should be considered an option because it may be beneficial in relieving both stridor and parkinsonian syndrome, and in preventing respiratory failure.

## 1 Introduction

Respiratory abnormalities are often overlooked; however, because of their potential comorbidity, their analysis is crucial for determining the most effective treatment for patients with Parkinson disease (PD). Stridor is one of the most serious of these conditions.^[1,2]^ Among numerous causes of stridor, vocal cord paralysis is frequently observed in multiple system atrophy, yet it has rarely been observed in patients with PD.^[3]^ Acute stridor often jeopardizes the safety of patients with PD. Without appropriate titration of antiparkinsonian drugs,^[4]^ respiratory failure can occur, or subsequent tracheostomy treatment is required. PD-related upper airway obstruction and restrictive respiratory difficulties usually improve after dopaminergic therapy,^[5,6]^ but levodopa-responsive stridor in PD is seldom reported.

## 2 Case report

**Figure 1:**
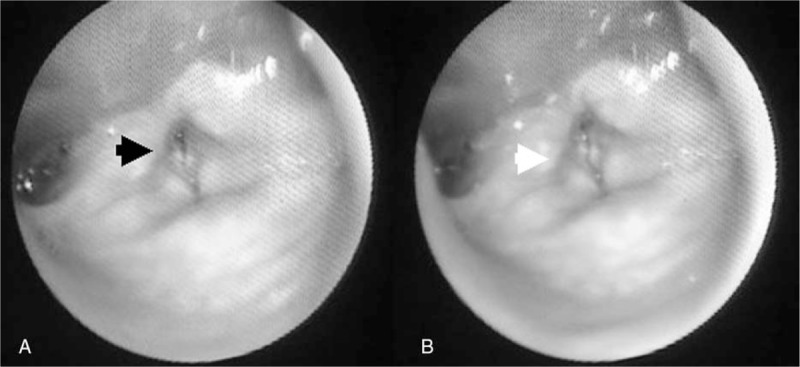
A, Bilateral vocal fold at paramedian position (black arrow) during inspiration. B, Bilateral vocal fold fixed at median position (white arrow) during expiration.

A 77-year-old gentleman with asymmetric and gradually progressive resting tremor, rigidity, and bradykinesia was diagnosed with PD 22 years ago. Through the treatment with Madopar HBS 125 mg, bromocriptine 2.5 mg, selegiline 2.5 mg, and biperiden 2 mg 2 times a day, his rigidity and bradykinesia symptoms insidiously deteriorated over the last 2 years of the disease period. The patient's disability level eventually reached stage 5 on the Hoehn and Yahr scale, and he was diagnosed with acute stridor with oxygen desaturation. No concurrent toxic signs were observed. The results of serological tests and imaging studies disclosed no unprecedented abnormalities. Flexible laryngoscope analysis performed by an otolaryngologist revealed the presence of bilateral vocal fold palsy (Fig. 1). Consequently, we up-titrated the patient's levodopa dosage to Madopar HBS 125 mg 4 times a day for 1 week. The patient's motor symptoms improved from stage 5 to stage 4 on the Hoehn and Yahr scale, and the stridor subsided; however, peak-dose dyskinesia subsequently occurred. After Madopar HBS was down-regulated to 3 times a day, dyskinesia was alleviated without stridor recurrence. Levodopa up-titration prevented the need for subsequent endotracheal intubation and tracheostomy treatment. No further respiratory distress was noted during the patient's admission.

## 3 Discussion

Among various theories of respiratory abnormalities in PD, “upper airway obstruction” and “restrictive respiratory disorders” are 2 of the most accepted etiologies; both appear to be related to basal ganglia dysfunction.^[1,6,7]^ Approximately 90% of PD patients suffer from dysphagia as the disease progresses. Both esophageal and laryngeal musculature are influenced by the nuclei ambigui, which are controlled by basal ganglia and its descending axons in the vagus nerve trunk.^[8]^ Dysfunction of the nuclei ambigui results in esophageal and laryngeal spasms. Reduced forced expiratory flow over a 1 second period in PD patients confirms upper airway obstruction.^[7]^ This concept is also supported by laryngeal electromyography and video-recorded fiberoptic endoscopy.^[7]^ Bradykinesia and rigidity of the respiratory muscles result in impaired performance of repetitive ventilation, engendering diminished strength of the respiratory pump muscles.^[2]^ Decreased peak expiratory flow and the maximum expiratory flow rate at 75% of vital capacity (maximum expiratory flow 75) in PD patients support this explanation.^[2]^ Respiratory function parameters and clinical PD severity are in correlation^[9]^ with each other.

Stridor is not only an abnormally high-pitched sound produced by turbulent airflow through airway, but also a lethal form of upper airway obstruction in PD patients. Bilateral vocal cord palsy, laryngeal spasms, and dystonia of the supralaryngeal muscle are its most frequent causes.^[10]^ Stridor can also be induced by localized disease in the laryngotracheal area, concurrent infection, or underlying metabolic disorders. The treatment of stridor is an essential component in preventing respiratory failure. Several previous studies have implied that levodopa administration induces considerable improvement in both lung function and parkinsonian syndrome.^[9]^ The outcome in our case may also suggest that respiratory abnormalities are possibly caused by nigrostriatal dopaminergic dysfunction. PD-related upper airway obstruction and restrictive respiratory difficulties usually improve with dopaminergic therapy.^[5,6]^ Gan et al defined the successful decannulation rate as being low in cases of bilateral vocal cord palsy, and higher following levodopa up-titration^[10,11]^; nevertheless, the crucial role of levodopa has not been specifically emphasized among suggested treatments.

After careful analysis of previous case studies and neurological examinations, we determined occurrence of multiple system atrophy to be less likely in our patient because of the following factors: the length of his disease (22 years), levodopa-induced dyskinesia, and improved motor performance after levodopa up-titration. The initial procedure for evaluating stridor should be analysis with flexible laryngoscopy. The resultant images of this procedure revealed bilateral vocal cord palsy in our patient. The amelioration of stridor through dopaminergic therapy suggested that bilateral vocal cord palsy could be a presentation of nigrostriatal dopaminergic dysfunction. Levodopa-induced respiratory dyskinesia can interfere with normal respiratory patterns, resulting in dyspnea, tachypnea, and irregular, erratic breathing patterns.^[6]^

## 4 Conclusion

In the present study, we reported a patient with PD and comorbid levodopa-responsive stridor resulting from bilateral vocal cord palsy. For the treatment of stridor, it is vital to understand that complex vocal cord muscle dysfunction may be related to nigrostriatal dopaminergic dysfunction in PD patients. The strategy of levodopa up-titration should be considered an option because it may be beneficial in relieving both stridor and parkinsonian syndrome, and in preventing respiratory failure.
